# Thalamic distribution and effects of 5-HT2C receptors on tonic GABAA inhibition and absence seizures: implications for treatment

**DOI:** 10.3389/fphar.2026.1729460

**Published:** 2026-02-20

**Authors:** Anna Cavaccini, Marcello Venzi, Cristiano Bombardi, Vincenzo Crunelli, Giuseppe Di Giovanni

**Affiliations:** 1 Neuroscience Division, School of Bioscience, Cardiff University, Cardiff, United Kingdom; 2 Laboratory of Neural Circuit Assembly, Brain Research Institute, University of Zurich, Zurich, Switzerland; 3 Department of Veterinary Medical Science, University of Bologna, Bologna, Italy; 4 Institute of Pharmacology and Neurosciences, Faculty of Medicine, University of Lisbon, Lisbon, Portugal; 5 College of Medicine, Korea University, Seoul, Republic of Korea; 6 Department of Medical and Surgical Sciences, University of Magna Graecia, Catanzaro, Italy

**Keywords:** GAERS rats, nucleus reticularis thalami, Ro 60-0175, SB242084, spike-and-wave discharges, thalamocortical neurons, ventrobasal complex

## Abstract

**Introduction:**

Childhood absence epilepsy (CAE) is associated with abnormal thalamocortical oscillations and enhanced GABAergic function in the ventrobasal (VB) thalamus, including increased extrasynaptic GABA_A_ receptor–mediated tonic currents in thalamocortical (TC) neurons. Serotonin signaling modulates seizure activity in several epilepsy models, and activation of 5-HT_2C_ receptors (5-HT_2C_Rs) has been reported to exert anti-absence seizure effects, although the underlying cellular mechanisms remain unclear. Here, we examined the thalamic distribution of 5-HT_2C_Rs and their functional impact on tonic GABAA inhibition and absence seizures.

**Methods:**

5-HT_2C_R expression in the nucleus reticularis thalami (NRT) and VB was assessed by immunohistochemistry in adult Wistar rats, Genetic Absence Epilepsy Rats from Strasbourg (GAERS), and their non-epileptic control strain (NEC). Whole-cell patch-clamp recordings were used to measure tonic GABAA currents in VB TC neurons in thalamic slices. In vivo EEG recordings in freely moving GAERS rats were performed to evaluate the effects of systemic administration of the 5-HT2CR agonist Ro 60-0175 on absence seizures.

**Results:**

No differences in 5-HT_2C_R expression were observed in the NRT across strains. In the VB, receptor expression was lowest in GAERS and highest in Wistar rats compared with NEC. Tonic GABAA currents in TC neurons were larger in GAERS than in Wistar or NEC rats. Activation of 5-HT_2C_Rs with Ro 60-0175 reduced tonic GABAA currents in TC neurons in all strains. Systemic administration of Ro 60-0175 in adult GAERS produced a clear reduction in absence seizures.

**Discussion:**

These findings indicate that 5-HT_2C_Rs regulate thalamic extrasynaptic GABAA inhibition and that their activation reduces tonic inhibitory drive in TC neurons while exerting anti-absence effects in vivo. The lower expression of 5-HT_2C_Rs in the GAERS VB suggests altered serotonergic control of thalamic inhibition in absence epilepsy. By reducing tonic GABAergic currents, 5-HT_2C_R activation may rebalance thalamocortical activity and suppress pathological oscillations, supporting these receptors as potential therapeutic targets for CAE.

## 1 Introduction

Childhood absence epilepsy (CAE) is characterized by brief non-convulsive seizures with spike-and-wave discharges (SWDs), arising from abnormal network oscillations (Crunelli and Leresche, 2002). Anatomical, electrophysiological, and computational studies indicate that these seizures emerge from dysfunctional interactions within the cortico–thalamo–cortical circuit, with additional contributions from basal ganglia networks (Vergnes et al., 1986; Hu and Wang, 2015; Depaulis et al., 2016; Hu et al., 2017; Jarre et al., 2017; Crunelli et al., 2020).

Thalamocortical (TC) neurons in the somatosensory ventrobasal thalamic complex (VB) exhibit phasic and tonic γ-aminobutyric acid type A (GABA_A_) currents (Belelli et al., 2005; Cope et al., 2005; Cope et al., 2009). Phasic inhibition depends on synaptic GABA_A_ receptors (GABA_A_R) (Cope et al., 2005) activated by GABA released from neurons of the nucleus reticularis thalami (NRT) (Belelli et al., 2005; Cope et al., 2005). In contrast, tonic GABA_A_ inhibition result from α4β2δ subunit-containing extrasynaptic GABA_A_Rs binding ambient GABA (Cope et al., 2005; Jia et al., 2005; Cope et al., 2009). This tonic current magnitude primarily depends on extracellular GABA levels which are mainly regulated by astrocytic GABA uptake and GABA release via glial transporters and bestrophin-1 (Cope et al., 2009; Kwak et al., 2020), though with a contribution from GABA release from the NRT (Herd et al., 2013).

Aberrant tonic GABA_A_ currents have been reported in different brain conditions including stress, stroke, traumatic brain injury, Parkinson’s disease and focal and generalized seizures, including absence seizures (ASs) (Belelli et al., 2009; Cope et al., 2009; Crunelli et al., 2012; Walker et al., 2012; Errington et al., 2014; Heo et al., 2020). These are genetic non-convulsive seizures that are characterized by brief lapses of consciousness with EEG SWDs (Huguenard, 2019; Crunelli et al., 2020; Deidda et al., 2021). We previously showed that in rodent models of ASs there is an enhanced tonic GABA_A_ current in VB TC neurons, due to a loss-of-function of the astrocytic GABA transporter 1 (GAT-1), which plays a key role in the expression of ASs (Cope et al., 2009; Errington et al., 2011; Crunelli et al., 2020; Deidda et al., 2021; Morais et al., 2021). This finding is supported by human data, showing that anti-seizure medicines (ASMs) that increase GABA levels can elicit and aggravate ASs (Perucca et al., 1998), and higher thalamic GABA levels were found in a child with ASs (Leal et al., 2016).

Thus, normalization of tonic GABA_A_ inhibition might have potential therapeutic value for rescuing ASs. Unfortunately, no selective antagonists for α4β2δ subunit-containing GABA_A_Rs have been synthesized yet. Thus, there is the need to modulate tonic GABA_A_ tonic inhibition indirectly, particularly through modulatory neurotransmitter systems. Among brainstem neurotransmitters modulating TC neuron excitability (McCormick, 1992; Di Giovanni et al., 2008; Crunelli and Di Giovanni, 2014; Deidda et al., 2021), serotonin (5-HT) is a key player, since it regulates cortico-thalamic activity (Varela and Sherman, 2009; Puig and Gulledge, 2011; Yang et al., 2015), impacting arousal, vigilance and cognition (Jacobs et al., 2000). The rodent thalamus receives dense and heterogeneous innervation from the raphe nuclei (Dahlstroem and Fuxe, 1964; Vertes et al., 1999; Rodriguez et al., 2011), with 5-HT receptors widely expressed in TC, NRT and thalamic interneurons in a nucleus-specific manner (Munsch et al., 2003; Li et al., 2004; Li et al., 2004; Bonnin et al., 2006; Coulon et al., 2010; Rodriguez et al., 2011). Additionally, 5-HT modulates thalamic GABA function (Bankson and Yamamoto, 2004; Crunelli and Di Giovanni, 2014; Crunelli and Di Giovanni, 2015; Goitia et al., 2016), and enhances GABA release (Deng and Lei, 2008).

5-HT modulates ASs in various rodent models (Jakus et al., 2003; Graf et al., 2004; Jakus et al., 2011; Russo et al., 2013; Deidda et al., 2021). Recently, we demonstrated altered 5-HT concentration in specific brain regions (De Deurwaerdère et al., 2022), and the anti-absence effect of activating 5-HT_2C_Rs in Genetic Absence Epilepsy rats from Strasbourg (GAERS) (Venzi et al., 2016), a well-validated model of ASs (Vergnes et al., 1982; Depaulis et al., 2016). However, the alteration of thalamic 5-HT_2C_R expression and the cellular mechanism responsible for the 5-HT_2C_R modulation of ASs remains unknown. Notably, 5-HT modulates tonic GABA_A_ currents in the visual thalamus (the dorsal lateral geniculate nucleus - dLGN) (Coulon et al., 2010; Crunelli and Di Giovanni, 2015), suggesting that the anti-absence effect of 5-HT_2C_R activation may depend on the ability of these receptors to modulate tonic GABA_A_ inhibition in VB thalamus.

Here, we directly tested this hypothesis by examining the distribution of 5-HT_2C_Rs in the VB and NRT using immunohistochemistry and by assessing the effects of 5-HT_2C_R activation on tonic GABA_A_ current in VB TC neurons of GAERS rats. Compared to outbred Wistar rats and. Because recent studies have shown that inbred non-epileptic control (NEC) rats display anatomical, neurochemical, and behavioral alterations despite the absence of seizures (Neuparth-Sottomayor et al., 2023; Casarrubea et al., 2024), we complemented the traditional GAERS/NEC comparison by including outbred Wistar rats, from which both strains are derived (Vergnes et al., 1982; Depaulis et al., 2016), as an additional reference strain. We found that 5-HT_2C_R expression was reduced in the GAERS VB compared to that of NEC and Wistar rats, while similar in the NRT among the three strains. Moreover, 5-HT_2C_R activation decreased tonic GABA_A_ inhibition *in vitro* and suppressed spontaneous ASs in freely moving GAERS rats, identifying these receptors as a promising therapeutic target.

## 2 Materials and methods

### 2.1 Experimental animals

All animal procedures were authorized by Cardiff University, in agreement with the United Kingdom Animals (Scientific Procedures) Act (1986) and international laws and policies (EU Directive, 2010/63/EU) for animal experiments. Male Wistar, GAERS and NEC rats were obtained from colonies bred at Cardiff University and housed at 21 °C under a 12 h light/dark cycle, with food and water available *ad libitum*. Immunohistochemistry and electrophysiological *in vitro* recordings were conducted on postnatal day (P) 25–30 rats, EEG recordings in P90-P120 rats. Every effort was made to minimize animal suffering and to reduce the number of animals used (Lidster et al., 2016).

### 2.2 Immunohistochemistry

Nine P25 rats (three NEC, three GAERS, three Wistar) were anesthetized (sodium pentobarbital 48 mg/kg, chloral hydrate 40 mg/kg, i.p.) and transcardially perfused with ice-cold saline (2 min, 30–35 mL/min) followed by 4% paraformaldehyde (30 min, 10 mL/min). Brains were post-fixed (3 h), cryoprotected in 30% sucrose (48 h, 4 °C), and sectioned coronally (30 μm) using a freezing microtome. Sections were stored in 30% sucrose/PBS (−20 °C) for immunohistochemistry or 10% formalin (RT) for thionin staining.

Free-floating coronal sections were washed in 0.02M PBS (3 × 10 min), treated with 1% H_2_O_2_ (15–30 min) to quench peroxidase activity, then rinsed (6×) in PBS. To block non-specific binding, sections were incubated (2 h, RT) in 10% normal goat serum (NGS, #CS 0922, Colorado Serum Co.) and 0.3% Triton X-100 in PBS. They were then incubated (48 h, 4 °C) with mouse anti-5-HT_2_CR antibody (1:100, sc-17797, Santa Cruz) in 1% NGS and 0.3% Triton X-100. Sections were washed (3 × 10 min) in PBS +2% NGS, then incubated (1 h, RT) with biotinylated goat anti-mouse IgG (1:200, BA-9200, Vector) in 1% NGS and 0.3% Triton X-100. After washing (3 × 10 min, PBS +2% NGS), they were transferred to an avidin–biotin complex (ABC kit, PK-6100, Vector) for 45 min, and the immunoperoxidase reaction was developed using a DAB kit (SK-4100, Vector). Sections were mounted on gelatin-coated slides, dried (overnight, 37 °C), dehydrated in ethanol, cleared in xylene, and coverslipped with Entellan (Merck, Darmstadt, Germany). For enhancement, sections were defatted, treated with OsO_4_ (0.005%, EMS #19130) and thiocarbohydrazide (0.05%, EMS #21900), then coverslipped with Entellan.

The mouse monoclonal anti-5-HT_2C_R antibody targets amino acids 374-458 of the human 5-HT_2C_R C-terminus. Its specificity was confirmed via Western blot and immunohistochemistry (Nocjar et al., 2015). In this study, control sections without primary antibodies showed no staining, and replacing or omitting secondary antibodies abolished immunostaining.

Adjacent sections were thionin-stained to delineate brain region boundaries. Sections were removed from 10% formaldehyde, mounted on gelatin-coated slides, and dried overnight at 37 °C. They were defatted in chloroform/ethanol (1:1) for 1 h, rehydrated through graded ethanol (100%, 96%, 70%, 50%; 2 min each), rinsed in dH_2_O (2 min), stained with 0.125% thionin (30 s), dehydrated, and coverslipped with Entellan (Merck, Darmstadt, Germany).

Sections were analyzed using a Leica DMRB microscope. Brightfield images were captured with a Polaroid DMC digital camera and DMC 2 software. Contrast and brightness were adjusted in Adobe Photoshop CS3. To assess 5-HT_2C_R-immunoreactive (IR) neuron density in the NRT and VB, immunostained somata were plotted in every fifth section using Accustage 5.1. Regional boundaries were outlined from adjacent thionin-stained sections with a stereo microscope and overlaid on computer-generated plots in Corel Draw X3. Area measurements were performed using AxioVision Rel.4.8. Neuron density (somata/mm^2^) was calculated separately for each section. Five sections per rat were analyzed, with counts expressed as mean ± SEM. Data were statistically analyzed using One-Way ANOVA with Tukey’s multiple comparisons. The Bonferroni test yielded similar results. Statistical significance was set at *P* < 0.05.

### 2.3 *In vitro* electrophysiological recordings and analysis

#### 2.3.1 Slice preparation

Acute horizontal brain slices containing the VB and the NRT were prepared as described previously (Cope et al., 2009). Briefly, acute horizontal brain slices (300 μm) containing the VB and the NRT were prepared from male P20-25 Wistar, GAERS and NEC rats, as described previously (Cope et al., 2005; Cope et al., 2009). During cutting, slices were maintained in a continuously oxygenated (95% O_2_: 5% CO_2_) ice-cold artificial cerebrospinal fluid (aCSF) containing (mM): 126 NaCl, 26 NaHCO_3_, 2.5 KCl, 2 MgCl_2_, 2 CaCl_2_, 1.25 NaH_2_PO_4_, 10 glucose, 0.045 indomethacin and 3 kynurenic acid. After cutting, slices were incubated in a chamber containing oxygenated aCSF of the above composition without indomethacin and kynurenic acid, for at least 1 h, before being transferred to the recording chamber. In the recording chamber, slices were continuously perfused (∼2 mL/min) with warmed (32 °C ± 1 °C) oxygenated recording aCSF of above composition, but with a lower concentration of MgCl_2_ (1 mM) and without indomethacin. Kynurenic acid was used in the cutting solution to increase slice viability and in the recording solution to block ionotropic glutamate receptors and therefore isolate GABA_A_R currents. Experiments were performed on only a single neuron for each slice.

#### 2.3.2 Electrophysiology

TC neurons of the VB were visualized using a Nikon (Tokyo, Japan) Eclipse E600FN microscope equipped with a 40x immersion lens and a video camera (Hamamatsu, Hamamatsu City, Japan). Whole-cell patch clamp recordings were made from VB TC neurons held at −70 mV using pipettes (resistance, 2.5–5 MΩ) containing (mM): 130 CsCl, 2 MgCl_2_, 4 Mg-ATP, 0.3 Na-GTP, 10 Na-HEPES and 0.1 EGTA, pH 7.25-7.30, (290–295 mOsm). Data were acquired using an Axopatch 200B amplifier controlled by pClamp 9.0 software (Molecular Device), filtered at 2 kHz and sampled at 20 kHz (Digidata 1322A; Molecular Device). Series resistance and whole-cell capacitance were determined from responses to 5 mV voltage steps. Series resistance was compensated by ∼80% and was continuously monitored during recordings. If the access resistance changed >20%, data were excluded from the analysis.

#### 2.3.3 Data collection, analysis and statistics

The tonic GABA_A_ current was measured as the outward change in baseline current observed following focal (via a pipette) application onto each slice of the GABA_A_ antagonist gabazine (100 μM, GBZ) (holding potential: −70 mV), as previously described (Rosenberg et al., 2017). In particular, the baseline current was measured as the averaged 20 s current before GBZ application. The shift in baseline current, measured as the averaged 20 s current after GBZ application, was compared to the baseline current. The average membrane capacitance of recorded VB TC neurons at P25–P30 was ∼100–120 pF, consistent with previous electrophysiological studies in juvenile GAERS and control rats (Cope et al., 2005; Cope et al., 2009). Tonic GABA_A_ current amplitude was normalized to whole-cell capacitance for each neuron. 5-HT_2C_R ligands were applied in the recording solution, either alone or in combination. Only one TC neuron was recorded in each slice, with each animal contributing two to three cells in total.

### 2.4 *In vivo* EEG recordings and analysis

EEG recordings were conducted on P90-P120 old male GAERS rats. The surgical procedure for chronic EEG implantation is described in detail elsewhere (Cope et al., 2009). Briefly, animals were anaesthetized with isoflurane and the body temperature maintained at 37 °C via a homoeothermic heat blanket (Harvard Apparatus, United States). Gold-plated EEG screw electrodes (Svenska Dentorama, Sweden) were implanted bilaterally over frontal and parietal cortices, and two ground electrodes were placed over the cerebellum. All electrodes were secured with acrylic cement. Rats were administered 0.5 mg/kg meloxicam post-operatively and were allowed to recover for at least 5 days before the start of the experiments.

On the day of the experiment the following drug administration protocol was used: rats were connected to the recording apparatus and left undisturbed for 1 h (habituation period), then the EEG and video recordings were started and a 40-min control period was recorded (control period). Afterwards, GAERS rats were randomly assigned to receive an intraperitoneal injection of either the selective 5-HT_2C_R agonist Ro 60-0175 (3 mg/kg, i. p.) (Orban et al., 2014; Di Giovanni and De Deurwaerdere, 2016; Silenieks et al., 2019) or its vehicle (0.1% of DMSO in saline), and the EEG and video recordings continued for an additional 2 h period (treatment period).

Data were acquired with a SBA4-v6 BioAmp amplifier (SuperTech Inc., Hungary), sampled at 1,000 Hz (Cambridge Electronic Design, CED, Micro3 D.130) and analyzed with CED Spike2 v7.3 and Matlab (R2014b, The Mathworks Inc., United States). Visual inspection and video confirmation were performed without knowledge of treatment condition. Detection of SWDs was performed as previously described (Cope et al., 2009). Briefly, the EEG data were DC removed and SWDs were detected semi-automatically using a custom-made Spike2 (SeizureDetect, Spike2, CED, Cambridge, United Kingdom). Detection was further refined by visual inspection of the data that was also used to confirm that the presence of motor arrest accompanied the EEG SWD, i.e., that an AS had occurred. Seizures were quantified using three parameters: total time spent in seizure, number of seizures and average seizure length (Marescaux et al., 1992). Values were normalized by expressing them as a percentage of the corresponding parameter in the control period (−40 to 0 min).

#### 2.4.1 Drugs

The 5-HT_2C_R agonist (2S)-1-(6-chloro-5-fluoroindol-1-yl)propan-2-amine (Ro 60-0175), the 5-HT_2C_R antagonist 6-chloro-5-methyl-N-(6-(2-methylpyridin-3-yl)oxypyridin-3-yl)-2 (SB242084) and gabazine (SR 95531 hydrobromide) were purchased from Tocris Bioscience (United Kingdom). The doses of Ro 60-0175 were chosen according to previous published works on epilepsy animal models and its general pharmacology to avoid off-target effects (Di Giovanni and De Deurwaerdere, 2016). General laboratory salts, indomethacin and kynurenic acid were purchased from Sigma Aldrich (United Kingdom).

#### 2.4.2 Statistics

Electrophysiological datawere analysed by one-way ANOVA (GraphPad Instat 3 software) followed by *post hoc* analyses (Dunnett’s and Dunn’s multiple comparison tests) if ANOVA yielded significant main effects. For comparisons of two groups, an independent two-sample t-test was used (GraphPad Instat 3 software). Significance was set at p < 0.05 for all statistical tests. All quantitative data are presented in the text and figures as mean ± SEM.

The anti-absence effects of the 5-HT_2C_R agonist in freely moving adult GAERS were assessed using a two-way repeated measures ANOVA. Treatment (drug vs. control) was included as the between-subjects factor, while time (1st vs. 2nd h) served as the within-subjects factor. To assess main effects and interactions on seizure frequency, Tukey’s *post hoc* test was applied.

## 3 Results

### 3.1 5-HT_2C_R-immunostaining in the NRT and VB of NEC, GAERS and Wistar rats

**FIGURE 1:**
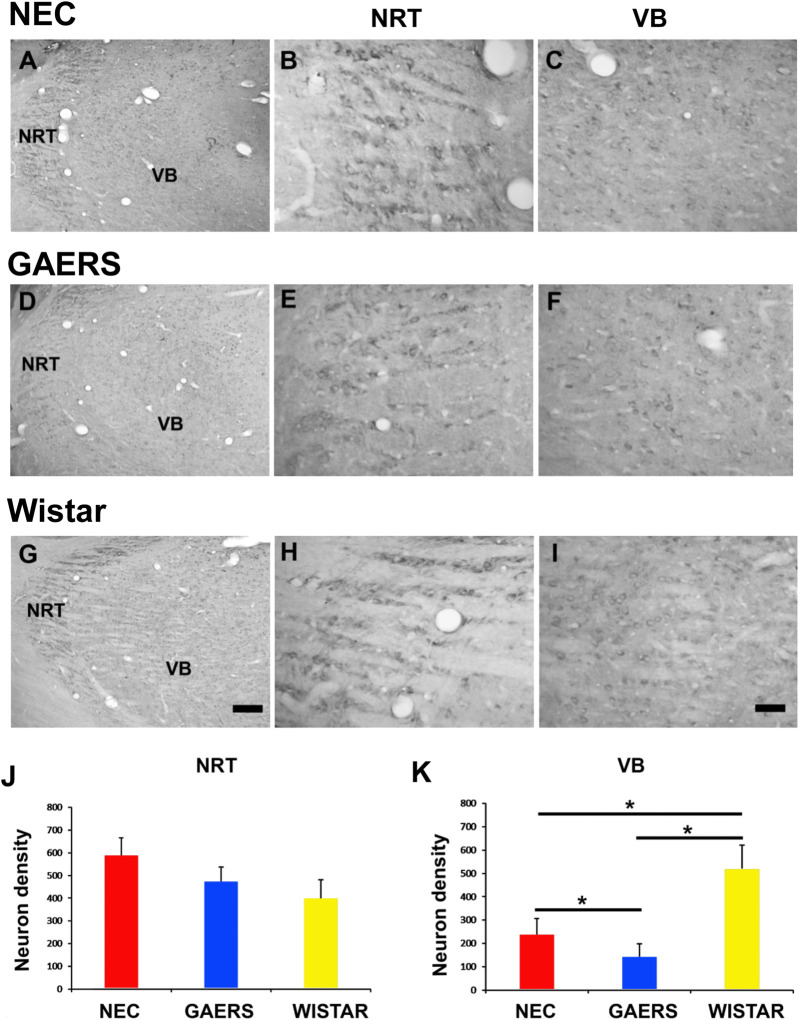
Distribution of 5-HT_2C_Rs in the VB and NRT. Brightfield photomicrographs showing the distribution of 5-HT_2C_ receptor immunoreactivity in the VB and NRT **(A,D,G)**, and enlarged sections of the NRT **(B,E,H)** and VB **(C,F,I)** of P25-30 NEC, GAERS and Wistar rats, as indicated. **(J,K)** Quantification of 5-HT_2C_R^+^ neuron density in the NRT and VB of the three strains. No significant differences were observed in the NRT. In the VB, Wistar rats displayed a significantly higher 5-HT_2C_R^+^ neuron density than NEC and GAERS (*P* < 0.05), with GAERS showing the lowest count (*P* < 0.05). Scale bar = 200 µm in **(G)** (applies to **(A,D,G)**) and = 50 µm in **(I)** (applies to **(B,C,E,F,H,I)**). One-Way ANOVA with Tukey’s multiple post-test comparison (*P < 0.05).

5-HT_2C_Rs are expressed in TC neurons across different thalamic nuclei with a nucleus-specific distribution (Munsch et al., 2003; Li et al., 2004; Coulon et al., 2010). In line with this evidence, 5-HT_2C_R immunostaining was detected in the somata and neuropil of the NRT and VB neurons in NEC, Wistar and GAERS. Neuropil immunoreactivity was diffuse, not associated with identifiable neuronal structure, and consistent among the three strains (Figures 1A,D,G). In the NRT, the density of 5-HT_2C_R-immunopositive (5-HT_2C_R^+^) neurons was unchanged across the three strains (Figures 1B,E,H–J). However, a one-way ANOVA revealed a significantly higher density of 5-HT_2C_R^+^ neurons in the VB of Wistar than NEC and GAERS rats (n = 3 for each strain, *P* < 0.05), with GAERS showing the lowest number of 5-HT_2C_R^+^ neurons (*P* < 0.05) (Figures 1C,F,I,K).

### 3.2 5-HT_2C_R modulation of the tonic GABA_A_ current in GAERS and NEC rats

**FIGURE 2:**
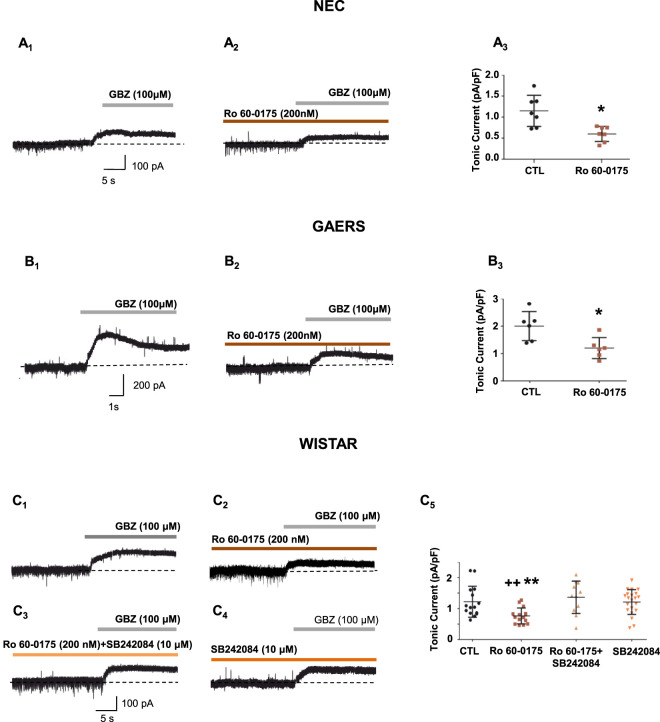
5-HT_2C_Rs modulate tonic GABA_A_ currents in Wistar, NEC and GAERS rats. **(A,B)** Representative current traces from different VB TC neurons in slices from NEC **(A**_**1,2**_ **)** rats and GAERS **(B**_**1,2**_ **)** under control condition **(A**_**1**_ **,B**_**1**_ **)**, and in the continuing presence of Ro 60-0175 **(A**_**2**_ **,B**_**2**_ **)**. The tonic GABA_A_ current is revealed by focal application of gabazine (GBZ) (see methods). Summary of the effects of Ro 60-0175 on the normalized tonic GABA_A_ current of NEC **(A**_**3**_ **)** and GAERS **(B**_**3**_ **)** (GAERS_CTL_: n = 6, GAERS_Ro 60–0175_: n = 6; *p < 0.05 Kruskall-Wallis ANOVA non parametric test, Dunn’s multiple comparisons test vs. CTL. NEC_CTL_: n = 7, NEC_Ro 60–0175_: n = 7, *p < 0.05 One-way Anova, Dunnett’s multiple comparisons test vs. CTL (see details in the text). **(C)** Representative current traces from different VB TC neurons in slices from Wistar rats under control condition **(C**_**1**_ **)**, and in the continuing presence of Ro 60-0175 **(C**_**2**_ **)**, Ro 60-0175 + SB242084 **(C**_**3**_ **)**, and SB242084 alone **(C**_**4**_ **)**. The tonic GABA_A_ current is revealed by focal application of gabazine (GBZ) (see methods). **(C**_**5**_ **)** Summary of the effects of Ro 60-0175, Ro 60-0175 + SB242084, and SB242084 alone on the normalized tonic GABA_A_ current (Wistar-CTL_Ro 60–0175_: n = 15, Wistar-Ro 60-0175: n = 15, Wistar-SB242084 +: n = 21, Wistar-SB242084: n = 11). **p < 0.01 vs. CTL; ^++^p < 0.01 vs. Ro 60-0175+SB242084, One-way Anova, Dunnett’s multiple comparisons test (see text for details).

To assess the potential translational significance of the above data for ASs, we next investigated whether the 5-HT_2C_R agonist Ro 60-175 was capable of reducing the aberrant tonic GABA_A_ current in VB TC neurons of GAERS (Venzi et al., 2016). We confirmed the presence of an enhanced tonic GABA_A_ current in epileptic GAERS (GAERS_CTL_: 2 ± 0.5 pA/pF, n = 6) compared to non-epileptic NEC (NEC_CTL_: 1.1 ± 0.4 pA/pF, n = 7, p < 0.01) (Figures 2A_1,3_, B_1,3_). Perfusion of slices with Ro 60–0175 significantly decreased (40% reduction) the tonic GABA_A_ current of GAERS (GAERS_Ro 60–0175_: 1.2 ± 0.4 pA/pF, n = 6, p < 0.05 vs. GAERS_CTL_) compared to that observed under control condition (Figures 2B_2,3_). Moreover, Ro 60–0175 significantly decreased (48% reduction) tonic GABA_A_ current in NEC rats (NEC_Ro 60–0175_: 0.6 ± 0.2 pA/pF, n = 7, p < 0.05 vs. NEC_CTL_) (Figures 2A_2,3_).

### 3.3 5-HT_2C_Rs modulate the tonic GABA_A_ current in Wistar rats

The selective 5-HT_2C_R agonist Ro 60–0175 significantly decreased (33% reduction) tonic GABA_A_ current of VB TC neurons (Wistar-RO 60-0175: 0.8 ± 0.3 pA/pF, n = 15) compared to control (Wistar-CTL_RO 60–0175_: 1.2 ± 0.5 pA/pF, n = 15; p < 0.01) (Figures 2C_1,2,5_). This inhibitory effect of Ro 60-0175 on the tonic GABA_A_ current was mediated by 5-HT_2C_Rs, since it was not present when the selective 5-HT_2C_R antagonist SB242084 (10 μM) was co-applied with Ro 60-175 in the recording solution (Wistar-SB242084 + Ro 60-0175: 1.2 ± 0.4 pA/pF, n = 21, vs. Wistar-Ro 60-0175, p < 0.01) (Figures 2C_3,5_). Moreover, perfusion of thalamic slices with SB242084 (10 μM) alone did not affect tonic GABA_A_ current compared to control (Wistar-SB242084: 1.4 ± 0.5 pA/pF, n = 11, vs. Wistar-CTL_RO 60–0175_, p > 0.05) (Figures 2C_4,5_), suggesting either the absence of a detectable basal 5-HT_2C_R tone on extrasynaptic GABA_A_R function or insufficient endogenous 5-HT levels *ex vivo* under our experimental conditions.

### 3.4 Systemic administration of Ro 60-0175 decreases ASs in freely moving adult GAERS rats

**FIGURE 3:**
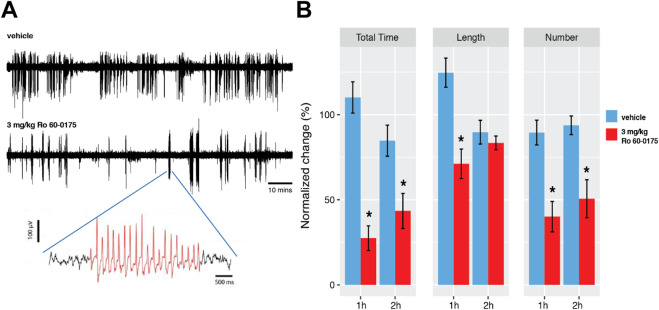
Effects of Ro 60–0175 on spontaneous ASs in GAERS rats. **(A)** Representative EEG traces from a GAERS rat injected i. p. either with vehicle (top trace) or 3 mg/kg Ro 60-0175 (bottom trace). An enlarged trace of a spike-and-wave discharge (SWDs) (red) is shown below. **(B)** Effect of Ro 60-0175 on total time spent in seizures, number of seizures and seizure length during the first and second h post-Ro 60-0175 treatment. All values are normalized to the control period (−40 to 0 min). Values represent mean ± SEM. Asterisks indicate *P* < 0.05 for Ro 60-0175 treatment vs. the corresponding time-bin in the vehicle group (two-way ANOVA, Sidak’s multiple comparison test) (n = 8 rats).

We have previously shown that lorcaserin and CP-809,101, two 5-HT_2C_R agonists, reduce spontaneous ASs in GAERS (Venzi et al., 2016). Since, i) a decrease in tonic current has an anti-absence effect (Cope et al., 2009), and ii) Ro 60-0175 reduces the tonic current in GAERS (Figure 2B_3_), we tested the effect of this 5-HT_2C_R agonist on spontaneous ASs recorded in freely moving adult GAERS. The systemic injection of Ro 60-0175 (3 mg/kg/i.p.) in eight GAERS rats reduced the total time spent in seizures (Figures 3A,B) (repeated measure two-way ANOVA on the time spent in seizure: *P* < 0.001; 62.8% reduction over the 2-h window). This anti-absence effect was driven by a 50.5% decrease in the number of seizures (Figure 3B) (p < 0.0001). The average seizure length, however, remained unchanged in the 2^nd^ hour of treatment (Figure 3B). To control for potential arousal or motor effects of systemic 5-HT_2C_R agonism (Di Giovanni et al., 2006; Di Giovanni and De Deurwaerdere, 2016), all seizure events were verified by simultaneous EEG and video recordings. These findings indicate that activation of 5-HT_2C_Rs by Ro 60-0175 exerts an anti-absence effect similar to that observed in the same rat model with lorcaserin and CP-809,101 (Venzi et al., 2016).

## 4 Discussion

In this study, we identified three key findings: 1) 5-HT_2C_R expression is lower in the VB, but similar in the NRT, of GAERS compared to Wistar and NEC rats; 2) 5-HT_2C_R activation reduces tonic GABA_A_ currents in TC neurons in Wistar, NEC and GAERS rats; 3) activation of 5-HT_2C_R by systemic Ro 60-0175 administration reduces ASs in freely moving GAERS, highlighting 5-HT_2C_Rs as potential therapeutic targets. Thus, our results provide *in vivo* and *in vitro* electrophysiological evidence that 5-HT_2C_Rs modulate tonic GABA_A_ inhibition in the somatosensory thalamus of both the healthy brain and those prone to the expression of spontaneous ASs. Moreover, our findings suggest that a strain-dependent differences in 5-HT_2C_R expression may contribute to altered thalamocortical inhibition in ASs, potentially underlying this seizure phenotype.

Inbred NEC rats have traditionally been used as a control strain for GAERS in studies of CAE (Depaulis et al., 2016; Crunelli et al., 2020), however, recent anatomical (Bombardi et al., 2018), neurochemical (De Deurwaerdère et al., 2022) and behavioral (Neuparth-Sottomayor et al., 2023; Casarrubea et al., 2024; Neuparth-Sottomayor et al., 2025) studies have revealed abnormalities in NEC rats, raising concerns about their suitability for comparative studies. For this reason, we have extended our invistigation to outbred Wistar rats. We previously found no functional differences in δ subunit-containing GABA_A_Rs among GAERS, NEC and Wistar rats, as the selective agonist THIP similarly increased tonic inhibition in all three strains (Cope et al., 2009). Despite GAERS rats exhibiting abnormally high tonic inhibition, attributed to the loss-of-function of GAT-1, its expression is similar in VB and cortex of GAERS and NEC (Cope et al., 2009). Accordingly, in this study we found that the tonic GABA_A_ current was higher in GAERS VB TC neurons and comparably lower in Wistar and NEC rats. Therefore, Wistar rats are comparable to NEC rats and may serve as controls for electrophysiological studies of the tonic GABA_A_ inhibition in GAERS.

Consistent with prior studies (Munsch et al., 2003; Li et al., 2004; Coulon et al., 2010), we observed that 5-HT_2C_Rs had a nucleus-specific distribution within the thalamus. No differences were observed in 5-HT_2C_R^+^ neuronal density within the NRT of the three strains. In contrast, GAERS rats exhibited a fivefold lower density of 5-HT_2C_R^+^ neurons in the VB compared to Wistar rats, with NEC rats significantly higher than GAERS but lower than Wistar. In line with an anticonvulsant 5-HT_2C_R role, a reduction 5-HT_2C_R expression has been associated with increased neuronal excitability in experimental temporal lobe epilepsy (Orban et al., 2014), and 5-HT_2C_R KO mice exhibit a higher susceptibility to convulsive seizures and occasionally die from epilepsy (Applegate and Tecott, 1998).

As expected, Ro 60-0175, reduced tonic GABA_A_ currents in Wistar, NEC and GAERS rats. The effect of 5-HT_2C_R activation in Wistar rats was blocked by co-application of SB242084, a selective 5-HT_2C_R antagonist (Di Matteo et al., 2000). This aligns with findings in visual dLGN, where mCPP, another 5-HT_2C_R agonist, decreased the tonic GABA_A_ current in an SB242084-dependent manner (Crunelli and Di Giovanni, 2015). Of note, SB242084 alone did not affect the tonic GABA_A_ current of VB TC neurons, suggesting either low endogenous 5-HT levels in our thalamic slices or the lack of basal 5-HT_2C_R modulation.

To test our hypothesis that the anti-absence effect of 5-HT_2C_R agonists observed *in vivo* (Venzi et al., 2016) is mediated by the normalization of the high tonic GABA_A_ inhibition in the thalamus, we used Ro 60-0175, a 5-HT_2C_R preferring agonist (Martin et al., 1998; Di Giovanni and De Deurwaerdere, 2016). Ro 60–0175 showed an anti-absence effect in freely moving adult GAERS, which was comparable to those of other 5-HT_2C_R agonists, such as CP-809,101 and lorcaserin in GAERS (Venzi et al., 2016) or mCPP in WAG/Rij (Jakus et al., 2003), another well-validated AS model (Coenen et al., 1992).

5-HT control of the tonic GABA_A_ inhibition varies by brain region and receptor subtype (Crunelli and Di Giovanni, 2015; Meis et al., 2018), but its intracellular pathways remain largely unexplored, except for its reduction in the visual cortex via a 5-HT_1A_/PKA mechanism (Jang et al., 2015). This effect is likely mediated by PKC phosphorylation of GABA_A_Rs (Connelly et al., 2013; Crunelli and Di Giovanni, 2014; Deidda et al., 2021). Indeed, inhibitors of PKC have been shown to block both the 5-HT_2_R-mediated suppression (in the prefrontal cortex (Feng et al., 2001) and enhancement (in the spinal cord (Xu et al., 1998; Li et al., 2000)) of phasic GABA_A_ currents. As shown in *Xenopus* oocytes expressing both 5-HT_2C_Rs and GABA_A_Rs (Huidobro-Toro et al., 1996), other calcium-dependent pathways, independent of protein kinases or phosphatases, may contribute to the inhibitory effects of 5-HT_2C_Rs on GABA_A_Rs seen in our study.

5-HT_2C_Rs are expressed on both NRT and VB TC neurons, therefore, their inhibitory modulation of the GABAergic transmission may involve pre and/or postsynaptic receptors on TC neurons. This completes a scenario in which 5-HT activation of 5-HT_1A_ and 5-HT_2A_Rs on presynaptic NRT terminals decreases and enhances VB GABA release, respectively (Goitia et al., 2016), while 5-HT_2C_Rs reduce GABAergic inhibition, highlighting the complexity of 5-HT modulation of thalamic functions. Our findings suggest that reduced VB 5-HT_2C_R levels, possibly due to increased levels of 5-HT, may lead to excessive tonic GABA_A_ current, promoting hypersynchronous oscillations in ASs. The Ro 60–0175 modulation of GABA_A_R inhibition likely underlies the anticonvulsant effect of the 5-HT_2C_R agonists *in vivo* in ASs (present evidence and (Venzi et al., 2016). Although the reduction of tonic GABA_A_ current by Ro 60-0175 is not disease-specific *per se*, its functional relevance is state-dependent, as only GAERS display an abnormally elevated tonic inhibition that can be normalized by 5-HT_2C_R receptor activation. Given the role of thalamocortical circuits in ASs (Cope et al., 2009; McCafferty et al., 2018) these findings highlight the serotonergic system’s role in thalamic excitability and AS ictogenesis. Furthermore, our study supports Wistar rats as a suitable control strain for GAERS studies (Neuparth-Sottomayor et al., 2023; Casarrubea et al., 2024).

Together with existing clinical evidence from Dravet syndrome and Lennox-Gastaut syndrome demonstrating antiseizure efficacy of serotonergic agents such as the 5-HT releaser fenfluramine (Sourbron and Lagae, 2023), 5-HT_2C_R agonist lorcaserin (Bialer and Perucca, 2022) and the selective 5-HT_2C_R superagonist LP352 (Ren et al., 2025), our mechanistic findings provide a strong rationale to evaluate serotonergic agents with 5-HT_2C_R engagement, in CAE, ideally in parallel with more selective 5-HT_2C_R agonists to disentangle target-specific effects. One limitation of our study, shared with much of the existing literature, is that *in vitro* patch-clamp experiments were performed in juvenile slices, as reliable whole-cell recordings from adult (P90–P120) thalamocortical neurons are technically unfeasible in standard acute slices and are therefore routinely conducted in P25–P30 (Cope et al., 2009). Notably, the pathological changes in the GAT1 transporter, that underlie the generation of ASs start from P17 and are maintained throughout adult life in GAERS rats (Morais et al., 2021). In contrast, robust expression of ASs in GAERS requires adult animals (≥P90) (Vergnes et al., 1986; Depaulis et al., 2016; Crunelli et al., 2020).

The study has some limitations, including modestly powered IHC datasets and reliance on prior antibody validation. Moreover, the dissociation between receptor density and function suggests that 5-HT_2C_R efficacy depends on localization, coupling, and circuit-level mechanisms rather than absolute receptor abundance.

In conclusion, a deeper understanding of the altered 5-HT_2C_R modulation of GABAergic inhibition in the thalamocortical loop and its underlying mechanisms could refine therapeutic strategies targeting 5-HT_2C_R pathways to restore inhibitory control and reduce ASs.

## Data Availability

The raw data supporting the conclusions of this article will be made available by the authors, without undue reservation.
